# Taxonomic distribution of SbmA/BacA and BacA-like antimicrobial peptide transporters suggests independent recruitment and convergent evolution in host–microbe interactions

**DOI:** 10.1099/mgen.0.001380

**Published:** 2025-04-16

**Authors:** Nicholas T. Smith, Amira Boukherissa, Kiera Antaya, Graeme W. Howe, Peter Mergaert, Ricardo C. Rodríguez de la Vega, Jacqui A. Shykoff, Benoît Alunni, George C. diCenzo

**Affiliations:** 1 Department of Biology, Queen’s University, Kingston, ON, K7L 3N6, Canada; 2 Department of Chemistry, Queen’s University, Kingston, ON, K7L 3N6, Canada; 3 Institute for Integrative Biology of the Cell, CNRS, CEA, Université Paris-Saclay, 91198, Gif-sur-Yvette, France; 4 Écologie Systématique et Évolution, Université Paris-Saclay, CNRS, AgroParisTech, 91198, Gif-sur-Yvette, France; 5 Université Paris-Saclay, INRAE, AgroParisTech, Institut Jean-Pierre Bourgin for Plant Sciences (IJPB), 78000, Versailles, France

**Keywords:** antimicrobial peptides, host–microbe interaction, molecular evolution, pathogenesis, peptide transport, rhizobium-legume symbioses

## Abstract

Antimicrobial peptides (AMPs) are often produced by eukaryotes to control bacterial populations in both pathogenic and mutualistic symbioses. Several pathogens and nitrogen-fixing legume symbionts depend on transporters called SbmA (or BacA) or BclA (BacA-like) to survive exposure to AMPs. However, how broadly these transporters are distributed amongst bacteria, and their evolutionary history, is poorly understood. We used computational approaches, including phylogenetic and sequence similarity analyses, to examine the distribution of SbmA/BacA and BclA proteins across 1,255 species spanning the domain *Bacteria*, leading to the identification of 71 and 177 SbmA/BacA and BclA proteins, respectively. *In vitro* sensitivity assays using legume AMPs and several BclA proteins confirmed that AMP transport is a common feature of BclA homologues. Our analyses indicated that SbmA/BacA homologues are encoded only by species in the phylum *Pseudomonadota* and are primarily found in just two orders: *Hyphomicrobiales* and *Enterobacterales*. BclA homologues are somewhat more broadly distributed and were found in clusters across four phyla. These included several orders of the phyla *Pseudomonadota* and *Cyanobacteriota*, the order *Mycobacteriales* (phylum *Actinomycetota*) and the class *Negativicutes* (phylum *Bacillota*). Many of the clades enriched for species encoding SbmA/BacA or BclA homologues are rich in species that interact with eukaryotic hosts in mutualistic or pathogenic interactions. These observations suggest that SbmA/BacA and BclA proteins have been repeatedly co-opted to facilitate associations with eukaryotic hosts by allowing bacteria to cope with host-encoded AMPs.

**Impact Statement**

Antimicrobial peptides (AMPs) produced by eukaryotes play important roles in controlling bacterial populations in a variety of host–microbe interactions. The ability of several bacterial species to tolerate exposure to a given AMP, and thus associate with specific eukaryotic hosts, depends on peptide transporters known as SbmA (also called BacA) or the BacA-like transporter BclA. To better understand the evolutionary history and roles of these AMP transporters in shaping the environmental distribution of bacteria, we used sequence analyses and functional assays to examine their distribution within the domain *Bacteria*. Surprisingly, SbmA/BacA homologues were identified only in the phylum *Pseudomonadota*. BclA homologues were distributed more broadly but nevertheless were limited to just four phyla. Notably, many clades enriched for SbmA/BacA or BclA homologues are rich in genera that interact with eukaryotic hosts, suggesting these proteins have been repeatedly co-opted to facilitate associations with eukaryotic hosts. We further observed that BclA proteins from distantly related organisms can share greater functional similarity than those from more closely related species, suggesting the function of these proteins is driven more by the niche of the organism than taxonomy.

## Data Summary

**Fig. 1.**
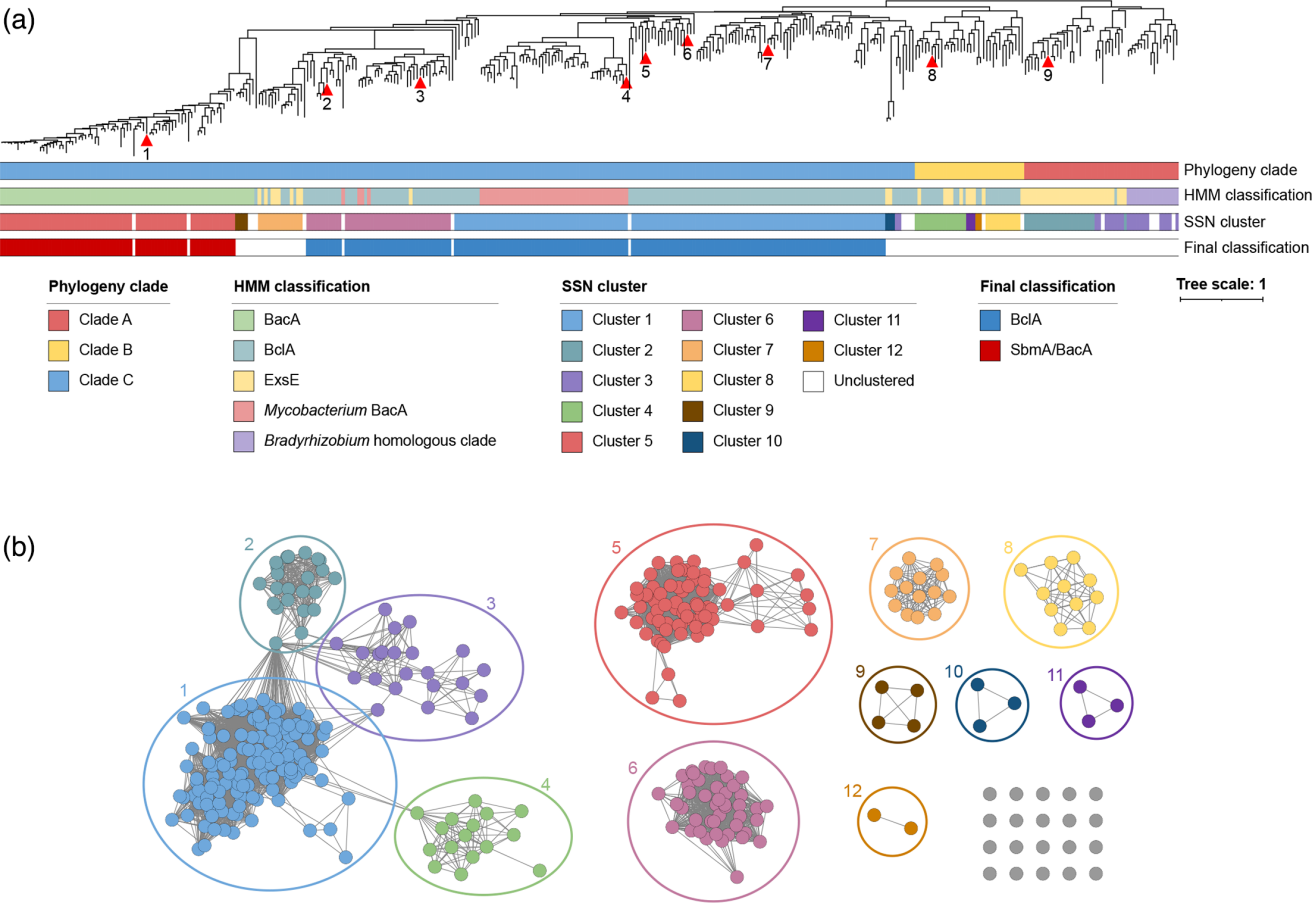
Sequence and phylogenetic analysis of SbmA/BacA-like proteins. (a) An unrooted maximum-likelihood phylogeny of 366 SbmA/BacA-like proteins is shown. The scale bar represents the average number of amino acid substitutions per site. Red triangles indicate proteins whose corresponding genes were codon optimized and synthesized: 1, *Polymorphum gilvum* BacA; 2, *Synechococcus elongatus* BclA; 3, *Cyanobacterium aponinum* BclA; 4, *Basilea psittacipulmonis* BclA; 5, *Succinivibrio dextrinosolvens* BclA; 6, *Methylomusa anaerophila* BclA; 7, *Polaromonas naphthalenivorans* BclA; 8, *Eikenella exigua* BclA-like; 9, *Phyllobacterium zundukense* ExsE (see Table 1 for more details). The bars beneath the phylogeny summarize the clustering and annotation of these proteins. The top bar indicates the phylogenetic clade to which each protein belongs. The second bar indicates the preliminary HMM classification of each protein. The third bar indicates the cluster in the SSN that each protein belongs to. The bottom bar indicates which proteins were ultimately classified as SbmA/BacA (red) or BclA (blue). An interactive version of this phylogeny, with node support values, is provided through iTol (https://itol.embl.de/shared/1IAjjFrHYGLI9) while a Newick-formatted version of the phylogeny can be downloaded from GitHub (https://github.com/amira-boukh/SbmA_BacA_phylogenetic_distribution). (b) An SSN, calculated using EFI-EST, of 366 SbmA–BacA-like proteins is shown. Each node (the circles) represents one protein, while edges (the lines) represent sequence similarity between pairs of proteins above the threshold, with longer lines indicating lower similarity. Nodes are colour coded based on cluster.

All genome sequences used in this work were previously published, and the assembly accessions are provided in Dataset S1. Likewise, all protein sequences included in Fig. 1 are provided in Dataset S2. Newick-formatted phylogenies used to create Figs. 1 and 4 are available through GitHub (https://github.com/amira-boukh/SbmA_BacA_phylogenetic_distribution). All code to repeat the analyses in this study is also available through GitHub (https://github.com/amira-boukh/SbmA_BacA_phylogenetic_distribution).

## Introduction

Bacteria, whether they thrive as free-living organisms or in interaction with eukaryotic hosts, are constantly challenged with a variety of stresses, including exposure to antimicrobial peptides (AMPs). These peptides are usually ca. 10–60 aa, and although they vary in their amino acid compositions, AMPs of the same family often display an enrichment in a specific amino acid, such as cysteine (forming intramolecular disulphide bridges), proline, arginine or glycine. These peptides may have two main modes of action depending on their cellular targets. Membrane-damaging AMPs interact with lipid bilayers and insert into biological membranes, thereby forming pores leading to cell content leakage and loss of ion gradients and membrane potential [1]. These are mostly cationic peptides, whose charge is involved in establishing an interaction with negatively charged microbial membranes, resulting in their destabilization. Other AMPs have intracellular targets and disturb metabolism and physiology, notably by interacting with metabolic enzymes, transcriptional and translational machineries and cell cycle regulators [2]. Bacteria have evolved several mechanisms to cope with AMPs, including the expression of dedicated AMP transporters [3,4].

An example of a bacterial transporter that can import AMPs is SbmA, which was originally identified in *Escherichia coli* as conferring sensitivity to the DNA gyrase inhibitor Microcin B17, a bacteriocin [5,6]. Homologues of SbmA (often known as BacA), and the related protein BclA (standing for BacA-like) [7], have since been identified in other bacteria and shown to be required for chronic infection of eukaryotic hosts by beneficial plant symbionts (e.g. nitrogen-fixing rhizobia) and by animal/human pathogens (*Brucella abortus* and *Mycobacterium tuberculosis*) [7,11]. SbmA/BacA and BclA are inner membrane peptide transporters, differing primarily by the presence of an ATPase domain in BclA that is absent in SbmA/BacA [7]. ATP hydrolysis by the ATPase domain is essential for the transport activity of BclA, whereas BacA-mediated transport is driven by the proton-motive force [12,13]. SbmA/BacA and BclA can import (and possibly export) a variety of AMPs, including nodule-specific cysteine-rich (NCR) peptides produced by some legume plants, proline-rich mammalian peptides and bacterial bacteriocins or microcins [5,7, 14,18]. They can also transport several non-proteinaceous compounds like the antibiotics gentamicin (Gm) and bleomycin, the vitamin cobalamin and possibly components of bacterial lipopolysaccharides [7,17, 19,21]. Another bacterial AMP transporter of note is the YejABEF complex. Like SbmA/BacA and BclA, YejABEF can import a range of AMPs and has been found in both plant mutualists and eukaryotic pathogens [22,25].

As many AMP transporters can import a range of AMPs, and considering the differing mechanisms of action of diverse AMPs, these transporters can have positive or negative effects on fitness depending on the environment. This is perhaps best demonstrated by considering the well-studied role of SbmA/BacA and BclA in rhizobia, which are bacteria capable of entering into nitrogen-fixing symbioses with legume plants. During these symbioses, rhizobia colonize a specialized legume root structure called a nodule, ultimately resulting in a chronic intracellular infection. Some legumes, like *Medicago truncatula*, produce a large family of cysteine-rich AMPs known as NCR peptides, whose isoelectric points vary from 3 (anionic) to 11 (cationic) [26,30]. These NCR peptides are involved in the control of rhizobial symbionts by mediating a process known as terminal bacteroid differentiation following their internalization by rhizobial cells in a SbmA/BacA or BclA-dependent fashion [30,31]. At the same time, strains of *Sinorhizobium meliloti* (a rhizobium) carrying loss-of-function *bacA* mutations are hypersensitive to cationic NCR peptide exposure *in vitro* [14] and die rapidly upon release into *M. truncatula* nodules in an NCR peptide-dependent fashion [9,14]. It has been hypothesized that by importing NCR peptides, BacA moves the cationic NCR peptides away from the cell membrane, thereby protecting *S. meliloti* from the membrane-damaging activities of these AMPs and promoting fitness [14,31, 32]. Although BacA may promote *S. meliloti* fitness during legume symbiosis, it may decrease fitness in the soil in the presence of AMPs produced by other microbes. Phazolicin, an AMP produced by the bacterium *Rhizobium* sp. Pop5, is toxic to other *Rhizobium* and *Sinorhizobium* strains due to its ability to inhibit translation intracellularly [33]. The import of phazolicin by *S. meliloti* is mediated by the BacA and YejABEF transporters (reducing fitness), with mutation of both transporters resulting in resistance to this AMP [18]. More broadly, a recent study predicted nearly one million new AMPs from microbiome data [34], suggesting that bacteria encounter many diverse AMPs in environmental niches. Thus, it is likely generally true that encoding AMP transporters like SbmA/BacA or BclA comes with a fitness trade-off, where these transporters promote fitness in the presence of membrane-targeting AMPs but impair fitness in the presence of AMPs with intracellular targets.

The observation that SbmA/BacA and BclA homologues are found in diverse bacterial lineages suggests that these proteins may be widespread housekeeping proteins subsequently co-opted for host–bacterial interactions [35]. On the other hand, the potential fitness trade-offs mean that the maintenance of these genes likely depends on the types of AMPs that a given bacterium encounters. However, no systematic study of the distribution of SbmA/BacA or BclA homologues across the bacterial tree exists. In addition, the evolutionary relationship between the SbmA/BacA and BclA families remains to be elucidated. Here, we report the distribution of SbmA/BacA and BclA homologues in 1,255 bacterial species from across the bacterial domain. We found SbmA/BacA homologues exclusively within the phylum *Pseudomonadota* (syn. *Proteobacteria*), while BclA homologues were predominately limited to the phyla *Pseudomonadota*, *Cyanobacteriota* (syn. *Cyanobacteria*), *Actinomycetota* (syn. *Actinomycetes*) and *Bacillota* (syn. *Firmicutes*). Expression of a subset of the newly identified BclA proteins in *S. meliloti* ∆*bacA* mutants confirmed that transport of AMPs is a common function of the BclA protein family. The taxonomic distribution of SbmA/BacA and BclA, together with phylogenetic analysis of these proteins, leads us to suggest that the functional similarities between SbmA/BacA and BclA are a result of convergent evolution and that these protein families have been repeatedly co-opted to help microbes cope with AMP exposure during host–microbe interactions and possibly in prokaryote–prokaryote interactions.

## Methods

### Bacterial strains and growth conditions

The bacterial strains used in this study are listed in Table S2. *E. coli* strains were cultured at 37 °C using lysogeny broth (LB; 10 g l^−1^ tryptone, 5 l^−1^ yeast extract, 5 g l^−1^ NaCl). *S. meliloti* strains were grown at 28 °C using either LBmc (LB supplemented with 2.5 mM CaCl_2_ and 2.5 mM MgSO_4_), yeast extract beef (YEB) (0.5% beef extract, 0.1% yeast extract, 0.5% peptone, 0.5% sucrose, 0.04% MgSO_4_ 7H_2_O, pH 7.5) or MM9 minimal medium (2% MOPS-KOH, 1.92% NH_4_Cl, 0.35% NaCl, 0.2% KH_2_PO_4_, 0.2% MgSO_4_, 0.05% CaCl_2_, 0.05% Biotin, 0.0004% CoCl_2_, 0.38% FeCl_3_, 1% Glucose, 1% Na_2_-succinate). Antibiotics were added as appropriate and included ampicillin (100 µg ml^−1^), kanamycin (Km; 100 µg ml^−1^), streptomycin (Sm; 200 or 500 µg ml^−1^), spectinomycin (Sp; 50 µg ml^−1^) and tetracycline (Tc; 5 µg ml^−1^). Antibiotic concentrations were generally halved for liquid cultures.

### Cloning of *bacA*, *bclA* and *exsE* homologues

Ten putative BacA, BclA or ExsE proteins were chosen as representatives from diverse clades within the protein phylogeny and to encompass different bacterial lifestyles (i.e. free-living vs. eukaryote-associated) (Fig. 1a and Table 1). Plasmids encoding each corresponding gene, codon optimized for *S. meliloti* 1021 and flanked by XbaI and BamHI recognition sites, were produced by Twist Biosciences (Table S2 and Dataset S1). Each gene was PCR amplified from the plasmids using Q5 polymerase (New England Biolabs; NEB) with the primers 5′-GAAGTGCCATTCCGCCTGACC and 5′-CACTGAGCCTCCACCTAGCC. The resulting amplicons were individually digested with XbaI/BamHI and ligated into XbaI/BamHI-digested expression vector pRF771 [36]. Plasmids were sequence verified via Illumina sequencing (151 bp paired-end reads) at SeqCenter (Pittsburg, PA, USA), after which reads were aligned to the expected template sequences using bowtie2 version 2.5.0 [37] and alignments visualized using the Integrative Genomics Viewer version 2.12.3 [38].

### Transfer of plasmids to *S. meliloti*

All plasmids of interest were transferred to an *S. meliloti* ∆*bacA* mutant via triparental matings using the helper strains *E. coli* MT616 or *E. coli* HB101 pRK600, as described previously [39,40]. Transconjugants were recovered through plating of mating spots on LBmc Sm^200^ Tc or YEB Sm^500^ Tc plates. Likewise, plasmids were transferred to an *S. meliloti* ∆*bacA* Ω*yejA* double mutant via triparental mating as described previously [39], with transconjugants recovered on YEB Sm^500^ Tc Km Sp plates. All transconjugants were streak purified three times prior to use.

### Gm sensitivity assays

Gm sensitivity assays were performed largely as described previously [32]. Briefly, overnight cultures of *S. meliloti*, grown in LBmc Sm^100^ Tc, were washed and resuspended in LBmc to an OD at 600 nm (OD600) of 1. In brief, 10 µl aliquots of the cell suspensions were added to triplicate wells of a 96-well plate and mixed with 190 µl of LBmc with or without 20 µg ml^−1^ of Gm. A Gm concentration of 20 µg ml^−1^ was chosen for the assays based on preliminary sensitivity assays (Fig. S4). Plates were tape-closed to prevent evaporation and then incubated at 30 ˚C with maximal shaking in a BioTek Synergy H1 plate reader for 24 h. OD600 measurements were collected every 15 min using the Gen5 software (Agilent Technologies).

### NCR247 sensitivity assays

NCR sensitivity assays were performed largely as described previously [23]. Briefly, overnight cultures of *S. meliloti*, grown in MM9 minimal media, were washed and resuspended in MM9 to an OD600 of 1. Cell suspensions were then diluted to an OD600 of 0.05, and 145 µl were transferred to the wells of 96-well plates and mixed with 5 µl of an NCR247 solution to reach final concentrations of 50, 25, 12.5, 6.25, 3.125 and 0 µg ml^−1^ of NCR247. Plates were incubated at 28 ˚C with shaking (180 r.p.m.) in a Tecan Spark plate reader for 72 h, and OD600 measurements were taken every 30 min and processed using the SparkControl software (Tecan).

### Plant symbiotic assays

Seeds of *M. sativa* cv. Algonquin (alfalfa) and *M. officinalis* (yellow-blossom sweet clover) (Speare Seeds Limited; Harriston, Ontario, Canada) were surface-sterilized and germinated on water agar plates for two nights in the dark, as described previously [27]. Leonard assemblies were prepared as described before [27], with a 1:1 (w/w) mixture of vermiculite and silica sand in the top pot, 250 ml Jensen’s medium [41] in the bottom pot and a cotton wick connecting the pots and then autoclaved. Five seedlings were sown per pot, and assemblies were incubated for two nights. Assemblies were next inoculated in triplicate with 1×10^8^ c.f.u. of *S. meliloti* per assembly. Plants were grown in a Conviron growth chamber with an 18 h photoperiod, 300 µmol s^−1^ of light, 21 °C daytime temperature and 17 °C nighttime temperature. After 30 days, plant shoots were collected and dried at 60 ˚C for six nights prior to weighing.

### Phylogenetic analysis of BacA and BclA proteins

GenBank files corresponding to 3,498 RefSeq bacterial genomes with ‘complete’ genome assemblies were downloaded from the National Center for Biotechnology Information (NCBI) Genome Database. A subset of the genomes was prepared by collecting genomes from one representative genome per genus, using the genome from the first species per genus when sorted alphabetically. The phylogenetic analyses were then repeated twice: once using all 3,498 RefSeq bacterial genomes and once using the reduced set of 1,255 genomes (Dataset S1). As the results were similar, we only present results generated using the reduced dataset.

BacA, BclA and related proteins were extracted from the bacterial proteomes using a modified version of an existing in-house pipeline [42]. The seed alignment of the SbmA/BacA-like family, consisting of eight sequences, was downloaded from Pfam (PF05992), and an HMM was built using the hmmbuild function of HMMER version 3.3 [43]. Separately, an HMM database was built by combining (i) the complete Pfam version 31.0 HMM database with (ii) the complete TIGERFAM version 15.0 HMM database, and then supplementing this database with HMMs of SbmA/BacA, BclA or related proteins that were built from diverse sources, including (iii) the seed alignments of PRK11098 (105 sequences in the seed alignment) and COG1133 (9 sequences in the seed alignment) downloaded from NCBI’s Conserved Domain Database and (iv) the BacA (15 sequences), BclA (5 sequences), *Mycobacterium* BacA (10 sequences), ExsE (6 sequences) and *Bradyrhizobium* homologous clade (7 sequences) proteins used in the phylogenetic analysis of Guefrachi *et al.* [7]. Next, the hmmsearch function of HMMER was used to search all bacterial proteomes using the PF05992 (SbmA/BacA-like family) HMM. All hmmsearch hits were then scanned against the full HMM database using the hmmscan function of HMMER. Each protein was annotated according to the top-scoring HMM from this search.

Proteins annotated as BacA, BclA, *Mycobacterium* BacA, ExsE or *Bradyrhizobium* homologous clade were extracted and aligned using Clustal Omega version 1.2.4 [44], hmmalign from HMMER [43] and MAFFT version 7.453 [45], and alignment quality assessed with T-COFFEE version 13.45 [46]. Poor-quality regions of the best-scoring alignment (clustal omega) were removed using trimAl version 1.4 with the automated1 option [47] and then used as input for maximum-likelihood phylogeny inference using IQ-TREE2 version 2.2.0 [48] with the LG+F+I+I+R9 model. The LG+F+I+I+R9 model was used, as it was identified as the best-scoring model by the IQ-TREE2 implementation of ModelFinder [49] based on Bayesian information criterion (BIC), with model search limited to the LG, WAG, JTT, Q.pfam, JTTDCMut, DCMut, VT, PMB, BLOSUM62 and Dayhoff models. Branch supports were assessed in IQ-TREE using the Shimodaira–Hasegawa-like approximate likelihood ratio test (SH-aLRT) [50] and an ultrafast bootstrap analysis, with both metrics calculated from 1,000 replicates. All phylogenies created in this study were visualized with the iTOL web server [51].

### SSN analysis

A SSN was constructed for the 366 proteins identified using the HMM approach described above. The SSN was constructed using the online enzyme function initiative’s enzyme similarity tool (EFI-EST; https://efi.igb.illinois.edu/efi-est/) [52,53] with an alignment score threshold of 115, corresponding to an approximate sequence ID≥35%. The resulting network was visualized using Cytoscape version 3.10.1 [54].

### Multilocus sequence analysis

A bacterial species phylogeny was produced for the 1,253 representative bacterial species using an adaptation of an existing in-house pipeline [42]; 2 of the 1,255 downloaded genomes were excluded as they encoded none of the marker genes. First, orthologs of 31 highly conserved, single-copy proteins (DnaG, Frr, InfC, NusA, Pgk, PyrG, RplA, RplB, RplC, RplD, RplE, RplF, RplK, RplL, RplM, RplN, RplP, RplS, RplT, RpmA, RpoB, RpsB, RpsC, RpsE, RpsI, RpsJ, RpsK, RpsM, RpsS, SmpB, Tsf) were identified in the 1,253 proteomes using the AMPHORA2 pipeline [55]. Each group of orthologs was individually aligned using MAFFT [45] and trimmed using trimAl with the automated1 option [47]. The protein alignments were then concatenated and used as input for ModelFinder as implemented in IQ-TREE2, and the best-scoring model was identified based on BIC. IQ-TREE2 was then used to infer a maximum-likelihood phylogeny from the concatenated alignment using the LG+I+I+R10 model. Branch supports were assessed in IQ-TREE using the SH-aLRT [50] and ultrafast jackknife analysis with a subsampling proportion of 40%, with both metrics calculated from 1,000 replicates.

## Results

### Identification and classification of SbmA/BacA and BclA homologues across the bacterial domain

To study the evolution and distribution of SbmA/BacA and BclA proteins, we searched the proteomes of 1,255 bacterial species, each belonging to a distinct genus, for proteins showing similarity to the SbmA/BacA-like family (Pfam: PF05992) (see section Methods). This process led to the identification of 366 putative SbmA/BacA-like family proteins from 258 species. We further classified each of these 366 proteins into one of five protein classes according to Guefrachi *et al.* [7]: SbmA/BacA, BclA, *Mycobacterium* BacA (a BclA-like family of proteins first identified in *M. tuberculosis*), ExsE (a related protein family involved in long-chain fatty acid transport) and the so-called *Bradyrhizobium* homologous clade (a related protein family with an unknown function). Initially, this classification was based on the use of hidden Markov models (HMMs), which was subsequently refined based on phylogenetic reconstruction and a sequence similarity network (SSN) as described below.

Using HMMs for these five protein classes, the 366 SbmA/BacA-like family proteins were initially classified into 79 SbmA/BacA proteins, 169 BclA proteins, 50 *Mycobacterium* BacA proteins, 52 ExsE proteins and 16 *Bradyrhizobium* homologous clade proteins (Fig. 1a). A maximum-likelihood phylogenetic analysis led to the identification of three primary monophyletic groups (Fig. 1a). Clade A comprised 48 proteins and included most ExsE and all *Bradyrhizobium* homologous clade proteins, which we treated as the outgroup. Clade B included 34 proteins that were annotated as a mix of BclA and ExsE based on the HMMs. Clade C was the largest clade, consisting of 284 proteins, and included most of the putative BclA, SbmA/BacA and *Mycobacterium* BacA proteins.

Most of the putative BclA proteins from Clade C also form a single cluster in the SSN (Cluster 1; Fig. 1b). We therefore conclude that the 133 proteins of Cluster 1 in the SSN represent true BclA homologues. Notably, Cluster 1 of the SSN also includes 46 proteins annotated as *Mycobacterium* BacA, which also fall within Clade C in the phylogeny (Fig. 1). This suggests that the *Mycobacterium* BacA proteins are not a distinct family from the BclA proteins and that *Mycobacterium* BacA proteins should instead be referred to as BclA. On the other hand, a Clade C subclade of nine proteins with long branch lengths in the phylogeny is excluded from Cluster 1 of the SSN; instead, two of these proteins are found as part of Cluster 3 that predominantly consists of the *Bradyrhizobium* homologous clade proteins, three are found as a three-protein cluster (Cluster 10) and five are singletons. In addition, four of these nine proteins are from strains encoding a BclA protein belonging to Cluster 1. Taken together, we conclude that these nine proteins are not true BclA homologues. Another subclade of Clade C consisting of 58 proteins is not part of Cluster 1 in the SSN but rather is largely found in two clusters (Clusters 6 and 7) of 44 and 14 proteins, respectively (Fig. 1). Cluster 6 consists primarily of proteins from cyanobacteria, and 43 of the 44 proteins were classified as BclA or *Mycobacterium* BacA by the HMMs. In addition, the functional data described below suggest that proteins of this cluster are functionally similar to known BclA proteins. We therefore conclude that proteins of Cluster 6 represent BclA homologues. In contrast, 8 of the 14 proteins of Cluster 7 were annotated as ExsE by the HMMs. The distinct clustering of Cluster 7 from Cluster 6, together with the HMM annotations, leads us to suggest that the proteins of Cluster 7 are unlikely to represent true BclA homologues.

Consistent with the phylogenetic analysis, proteins of Clade B do not cluster with proteins of Clade C in the SSN (Fig. 1b). Rather, the Clade B proteins are split across four clusters and two singletons. Nearly one-third (10 of 34) of the Clade B proteins were annotated as ExsE by the initial HMM strategy, and many of the proteins of Clade B are from bacterial strains that also encode a putative SbmA/BacA or BclA of Clade C. Collectively, we interpret these results to indicate that Clade B proteins are not part of the BclA protein family and that they instead represent a related but distinct protein family. This conclusion is also supported by the functional data presented below.

Finally, all putative SbmA/BacA proteins formed a monophyletic group in the phylogeny (Fig. 1a) and a monophyletic group of 71 of the 79 proteins forms a single cluster (Cluster 5) in the SSN (Fig. 1b). These results suggest that the 71 proteins of Cluster 5 that were annotated as SbmA/BacA by the HMM strategy are likely true SbmA/BacA homologues and that all SbmA/BacA proteins evolved from a common ancestor. Although the SbmA/BacA proteins fell within Clade C in the phylogeny, the SbmA/BacA clade is connected to the rest of the tree via a notably long branch, consistent with the distinct clustering of SbmA/BacA proteins in the SSN, as well as the functional differences in the transport of SbmA/BclA proteins (proton-driven) compared to BclA proteins (ATP-driven).

In considering the different sources of information described above, we ultimately chose to select a final set of SbmA/BacA and BclA proteins based primarily on the SSN, resulting in the identification of 177 high-confidence BclA proteins (including the *Mycobacterium* BacA proteins) and 71 high-confidence BacA proteins (Fig. 1a).

### *In vitro* functional analysis of diverse SbmA/BacA and BclA homologues

To validate that the BclA and SbmA/BacA proteins identified through the *in silico* approach are functionally similar to known BclA and SbmA/BacA proteins, we synthesized genes encoding nine of the identified proteins, collectively representing the diversity of sequences based on the SbmA/BacA and BclA phylogeny and coming from bacteria with diverse lifestyles (Table 1 and Fig. 1a). The proteins encoded by these genes included the following: one BacA protein, six BclA proteins including one previously classified as *Mycobacterium* BacA, one protein from Clade B (henceforth referred to as BclA-like) and one ExsE protein for comparison. The nine genes were then cloned into an expression vector and introduced into *S. meliloti* ∆*bacA* and *S. meliloti* ∆*bacA* Ω*yejA* mutants to test for complementation. Although the genes were codon optimized for expression in *S. meliloti*, we cannot exclude the possibility that some proteins were not properly expressed or were not stably inserted into the *S. meliloti* inner membrane. Therefore, lack of complementation may reflect improper expression/localization of a protein rather than a lack of functional similarity. All strains showed similar growth in media lacking antimicrobial agents (Fig. S1, available in the online Supplementary Material), indicating that differences in media supplemented with Gm or NCR peptides reflect altered resistance phenotypes rather than general growth differences. In addition, we observed that the resistance phenotypes of the *S. meliloti* ∆*bacA* mutant complemented with the *S. meliloti bacA* gene *in trans* differed somewhat from wild-type *S. meliloti* (Fig. S2), likely due to elevated expression of *bacA* in the complemented strain. Thus, for all *in vitro* phenotypic experiments, strains were compared to the *S. meliloti* ∆*bacA* mutant complemented with the *S. meliloti bacA* gene *in trans* rather than the wild type.

**Table 1.** SbmA/BacA, BclA and related genes synthesized experimentally characterized in this study

Protein no.	Species of origin	Order (Phylum)	Lifestyle (extracted from BacDive)*	Finalclassification	Phylogeny clade	HMMclassification	SSN cluster
1	“*Polymorphum gilvum*”	*Rhodobacterales* (*Pseudomonadota*)	Free-living oil-degrading	BacA	C	BacA	5
2	*Synechococcus elongatus*	“*Synechococcales*” (*Cyanobacteriota*)	Free-living photosynthetic (isolated from freshwater)	BclA	C	BclA	6
3	*Cyanobacterium aponinum*	*Chroococcales* (*Cyanobacteriota*)	Free-living photosynthetic (isolated from freshwater)	BclA	C	BclA	6
4	*Basilea psittacipulmonis*	*Burkholderiales* (*Pseudomonadota*)	Eukaryote-associated (isolated from dead bird lung)	BclA	C	M-BacA^†^	1
5	*Succinivibrio dextrinosolvens*	*Aeromonadales* (*Pseudomonadota*)	Eukaryote-associated (isolated from rumen of cow)	BclA	C	BclA	1
6	*Methylomusa anaerophila*	*Selenomonadales* (*Bacillota*)	Free-living (isolated from microbial fuel cell)	BclA	C	BclA	1
7	*Polaromonas naphthalenivorans*	*Burkholderiales* (*Pseudomonadota*)	Free-living (isolated form coal-tar contaminated site)	BclA	C	BclA	1
8	*Eikenella exigua*	*Neisseriales* (*Pseudomonadota*)	Eukaryote-associated (isolated from brain abscess and blood)	BclA-like	B	BclA	4
9	*Phyllobacterium zundukense*	*Hyphomicrobiales* (*Pseudomonadota*)	Eukaryote-associated (isolated from plant root nodules)	ExsE	A	ExsE	2
– ^‡^	*Bradyrhizobium* sp. ORS285	*Hyphomicrobiales* (*Pseudomonadota*)	Eukaryote-associated (isolated from plant root nodules)	BclA	–	–	–
– ^‡^	*Sinorhizobium meliloti*	*Hyphomicrobiales* (*Pseudomonadota*)	Eukaryote-associated (isolated from plant root nodules)	BacA	–	–	–

* Data extracted from the BacDive (ref) [75] entry for the type strain of each species. However, free-living organisms may also be host-associated even if we have no current evidence.

† M-BacA is short for *Mycobacterium* BacA.

‡ These proteins were included in the experimental characterizations not based on the analyses reported in Fig. 1, but rather as examples of previously characterized, known BclA and BacA proteins.

**Fig. 2.**
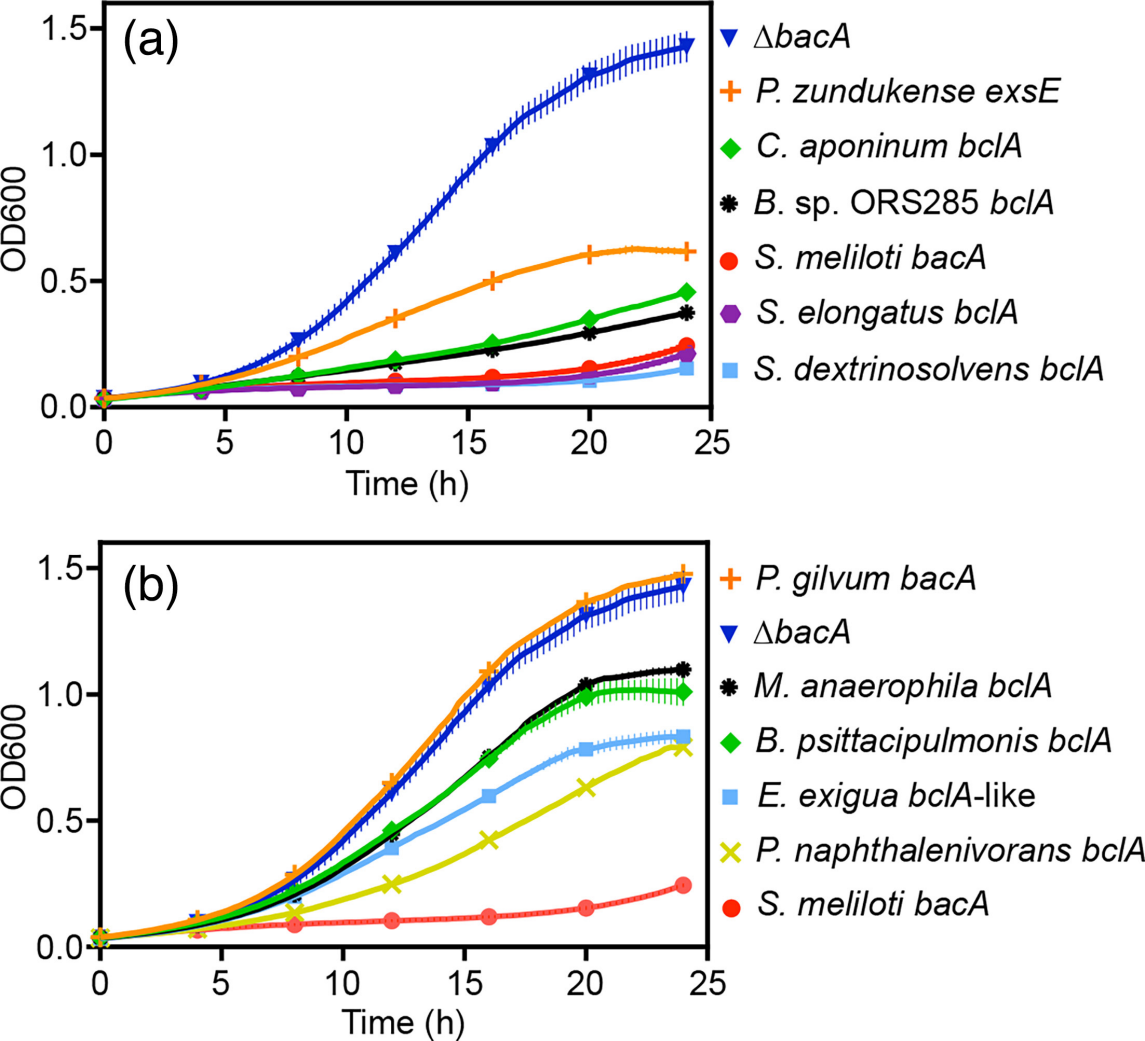
Gm sensitivity assays. The growth of various *S. meliloti* strains, as measured by OD600, in the presence of 20 µg ml^−1^ of Gm is shown over a 24 h period. Each point represents the mean of triplicate wells, with error bars depicting sd. The ∆*bacA* strain represents the *S. meliloti* ∆*bacA* mutant carrying an empty vector, while all other strains are named according to the species of origin of the gene expressed *in trans* in the *S. meliloti* ∆*bacA* background. The experiment was replicated three independent times, and data from a representative experiment are shown. (a) Data are shown for genes exhibiting moderate to high level of complementation of the *S. meliloti* ∆*bacA* Gm resistance phenotype. (b) Data are shown for genes exhibiting low to moderate levels of complementation of the *S. meliloti* ∆*bacA* Gm resistance phenotype.

As *S. meliloti bacA* null mutants display increased resistance to Gm [20], we first tested whether the nine genes could complement the Gm resistance phenotype of the *S. meliloti* ∆*bacA* mutant. As expected, the ∆*bacA* mutant was resistant to Gm, and re-introduction of the *S. meliloti bacA* gene *in trans* resulted in sensitivity to Gm (Fig. 2a). Introduction of the *Phyllobacterium zundukense exsE* gene resulted in intermediate complementation of the Gm resistance phenotype (Fig. 2a), suggesting that transport of Gm is a broadly conserved function of SbmA/BacA and related proteins and is not specific to BclA or SbmA/BacA proteins. As a result, the impact of the nine genes on Gm resistance cannot be used to support the annotation of a protein specifically as BclA or SbmA/BacA; however, it is still a useful metric to test whether a SbmA/BacA-like protein is expressed and functional. Of the six *bclA* genes identified by our screen, three (from *Cyanobacterium aponinum*, *Synechococcus elongatus* and *Succinivibrio dextrinosolvens*) complemented the Gm resistance phenotype at least as well as the known *bclA* gene of *Bradyrhizobium* sp. ORS285 (Fig. 2a), confirming they are expressed and functional in *S. meliloti*. The other three *bclA* genes all displayed partial complementation to varying degrees (Fig. 2b), suggesting they are expressed and functional but either have reduced ability to transport Gm or their expression or stability is sub-optimal. Likewise, the one BclA-like gene (from *Eikenella exigua*) displayed partial complementation of the Gm resistance phenotype (Fig. 2b). On the other hand, the introduction of the one *bacA* gene that we tested (from “*Polymorphum gilvum*”) completely failed to complement the Gm resistance phenotype of the *S. meliloti* ∆*bacA* mutant (Fig. 2b); based on protein structural comparisons (not shown), we hypothesize this result is due to improper expression or stability of the “*P. gilvum*” protein rather than functional divergence.

**Fig. 3.**
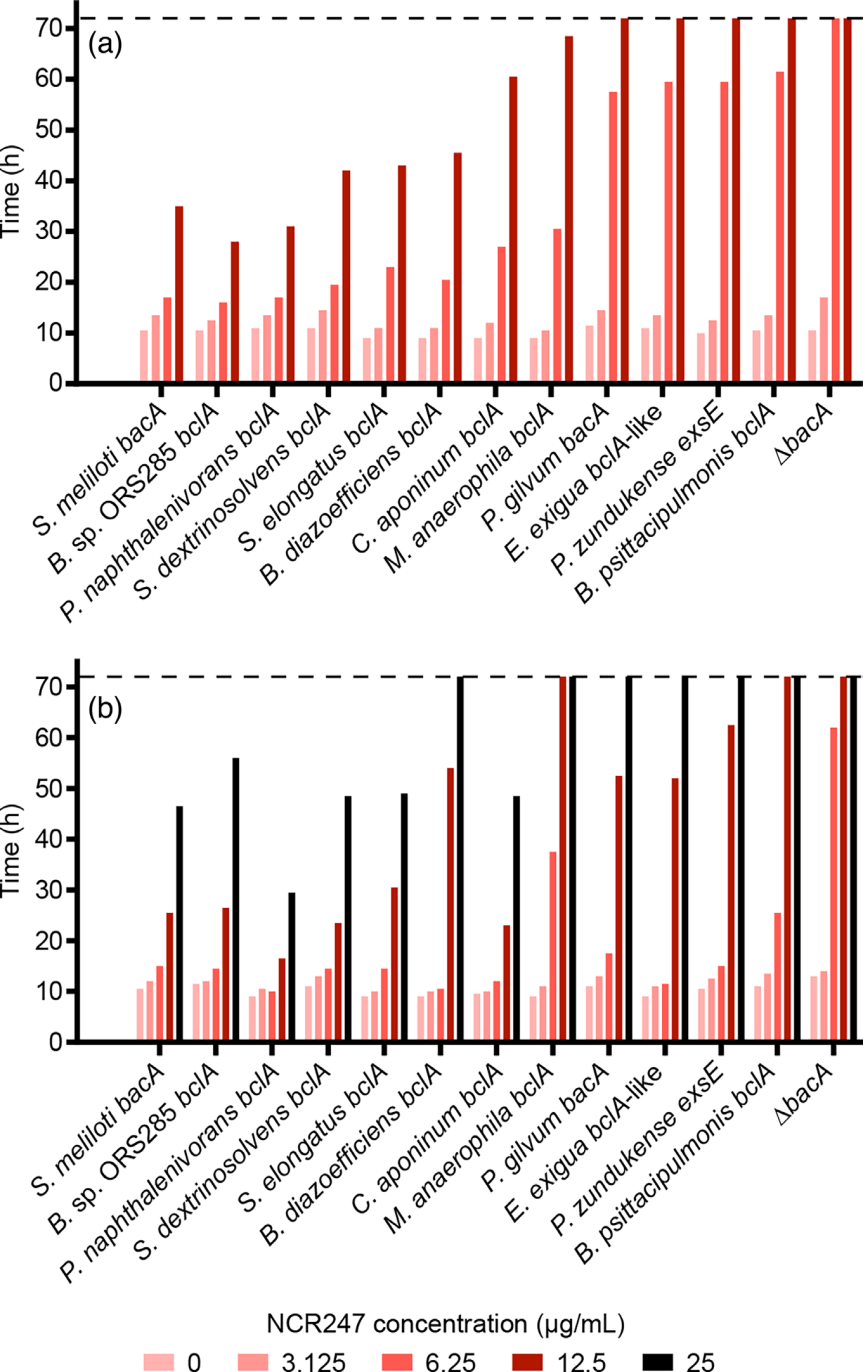
NCR247 sensitivity assays. The growth of various *S. meliloti* strains, as measured by OD600, in the presence of the AMP NCR247 is shown. Strains were grown in various concentrations of NCR247 as indicated by the shade of red or black. Bars represent the time required for the culture to reach an OD600 of 0.25. Values of 72 h (indicated by the dashed line) indicate that the strain failed to reach an OD600 of 0.25 within the 72 h growth period. The ∆*bacA* label represents the *S. meliloti* (a) ∆*bacA* or (b) ∆*bacA* Ω*yejA* mutant carrying an empty vector, while all other strains are named according to the species of origin of the gene expressed *in trans* in the *S. meliloti* (a) ∆*bacA* or (b) ∆*bacA* Ω*yejA* background. (a) Data are shown for the *S. meliloti* ∆*bacA* mutant and derivatives. (b) Data are shown for the *S. meliloti* ∆*bacA* Ω*yejA* mutant and derivatives.

We next indirectly examined whether the nine proteins could transport eukaryotic AMPs by measuring the impact of the proteins on the sensitivity of *S. meliloti* to the legume-encoded cationic NCR peptide NCR247 (Fig. 3). We selected NCR247 as a model AMP, as it was first synthesized by Van de Velde *et al.* [30] and has since become the most widely studied NCR peptide. This peptide crosses the *S. meliloti* cell envelope to interact with intracellular targets [31] and is notably capable of sequestering haem, promoting iron uptake and upregulating nitrogenase-catalysed nitrogen fixation in rhizobia [56]. The NCR247 peptide also forms pores in bacterial cell envelopes, resulting in cell death, and it is thought that proteins capable of importing NCR247 reduce its ability to damage the membrane and thus reduce sensitivity to this peptide [14,30]. Accordingly, the *S. meliloti* ∆*bacA* single mutant and the ∆*bacA* Ω*yejA* double mutant were hypersensitive to NCR247 exposure, while introduction of the known *S. meliloti bacA* or *Bradyrhizobium* sp. ORS285 *bclA* genes *in trans* resulted in reduced sensitivity to NCR247 (Fig. 3). Introduction of the *P. zundukense exsE* gene into the two mutants resulted in little to no complementation of the NCR247 hypersensitivity phenotypes (Fig. 3), consistent with the transport of NCR peptides being specific to proteins of the SbmA/BacA and BclA families and not a general property of these and related proteins. All three of the *bclA* genes showing strong complementation of the Gm resistance phenotype (two of which are from cyanobacteria) also showed good complementation of the NCR247 hypersensitivity phenotype (Fig. 3), confirming the proteins encoded by these three genes are functionally similar to known BclA proteins. In addition, the *bclA* gene from *Polaromonas naphthalenivorans* strongly complemented the NCR247 hypersensitivity phenotypes of both strains despite only moderate complementation of the Gm resistance phenotype. Of the remaining two *bclA* genes, one (from *Methylomusa anaerophila*) displayed weak complementation of the NCR247 hypersensitivity (Fig. 3) and varied in its level of complementation across trials (not shown), while one (from *Basilea psittacipulmonis*) failed to complement (Fig. 3). Overall, the data for the six BclA proteins suggest that most BclA proteins are capable of transporting NCR peptides. On the other hand, the NCR247 sensitivity phenotypes of the strains expressing the BclA-like protein from *E. exigua* resembled the phenotypes of the strains expressing *P. zundukense exsE* (Fig. 3), consistent with BclA-like proteins of Clade B (Fig. 1) representing a different class of proteins from BclA. In accordance with the Gm resistance data, the *bacA* gene from *P. gilvum* largely failed to complement the NCR247 hypersensitivity phenotypes (Fig. 3), potentially reflecting improper expression or stability of the encoded protein.

### Analysis of the ability of BacA and BclA to support legume symbiosis

We additionally tested whether the nine proteins could complement the nitrogen-fixation defect of an *S. meliloti* ∆*bacA* mutant during symbiosis with *Medicago sativa* (alfalfa) or *Melilotus officinalis* (yellow-blossom sweet clover). As expected, the *S. meliloti* ∆*bacA* mutant formed small white nodules on both plants and failed to fix nitrogen, while re-introduction of the *S. meliloti bacA* gene *in trans* complemented the nitrogen-fixation phenotype (Table S1). All nine of the synthesized genes failed to complement the nitrogen-fixation phenotype (Table S1). As the same lack of complementation was observed for the known *bclA* gene of *Bradyrhizobium* sp. ORS285 (Table S1), these results suggest that most, if not all, BclA proteins are unable to support an effective symbiosis between *S. meliloti* and its host plants. This is consistent with previous work showing that most *bacA* and *bclA* genes are unable to restore nitrogen fixation when expressed in an *S. meliloti bacA* null mutant [7,32, 39, 57], suggesting that SbmA/BacA and BclA homologues display slight variations in their peptide substrate range or rate of transport [27].

### Taxonomic distribution of SbmA/BacA and BclA homologues across the domain *Bacteria*

**Fig. 4.**
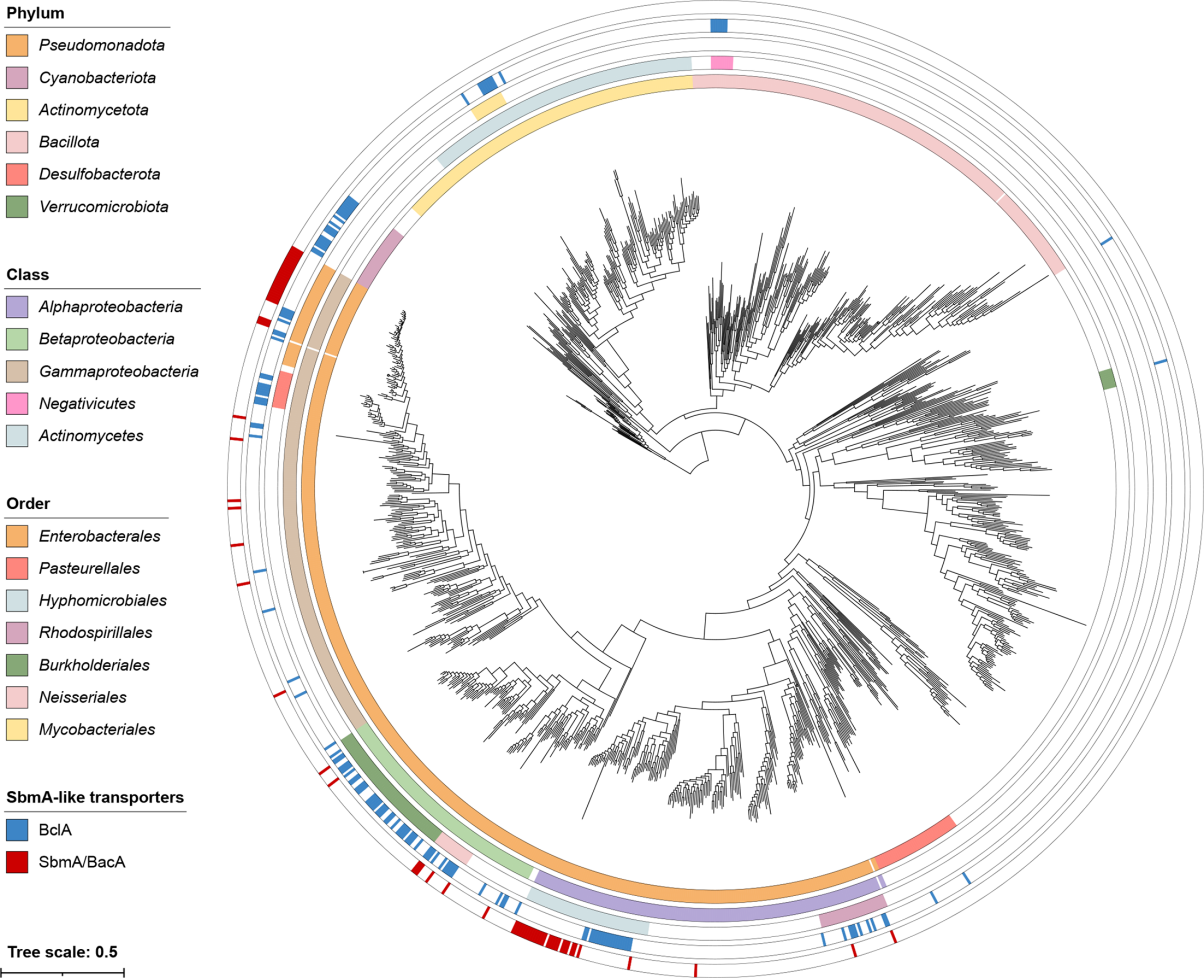
Taxonomic distribution of SbmA/BacA and BclA proteins in the domain *Bacteria*. An unrooted maximum-likelihood phylogeny of 1,533 bacteria is shown, inferred from the concatenated protein alignments of 31 single-copy proteins. The scale bar represents the average number of amino acid substitutions per site. Three clades of intracellular symbionts/pathogens with long branch lengths were removed for presentation purposes; none of these taxa encode SbmA/BacA or BclA. The rings represent the following, starting from the inner ring: (i) the phylum that each strain belongs to, limited to phyla where at least one strain encodes SbmA/BacA or BclA; (ii) the class that each strain belongs to, limited to classes where at least one strain encodes SbmA/BacA or BclA and that are mentioned in the text; (iii) the class that each strain belongs to, limited to classes where at least one strain encodes SbmA/BacA or BclA and that are mentioned in the text; (iv) whether the strain encodes BclA (blue) or not (white); (v) whether the strain encodes SbmA/BacA (red) or not (white). An interactive version of this phylogeny, with node support values and without collapsing of any clades, is provided through iTol (https://itol.embl.de/shared/1IAjjFrHYGLI9) while a Newick-formatted version of the phylogeny can be downloaded from GitHub (https://github.com/amira-boukh/SbmA_BacA_phylogenetic_distribution).

We next examined the taxonomic distribution of the 177 BclA and 71 SbmA/BacA proteins identified as described earlier. Remarkably, 100 and 78% of the identified SbmA/BacA and BclA proteins, respectively, are encoded by species of the phylum *Pseudomonadota* (syn. *Proteobacteria*) (Fig. 4). As expected, most species encoding SbmA/BacA or BclA proteins encode only one or the other; only 6 of the 208 species encoding SbmA/BacA and/or BclA encode both, and in all 6 cases, both genes are carried by the chromosome.

In brief, ~70% of the SbmA/BacA proteins are encoded by just two monophyletic groups of organisms, suggesting that SbmA/BacA was acquired at the base of each clade and then vertically transmitted. These two clades are a 24 species clade in the order *Enterobacterales* (all of which encode SbmA/BacA) and a 29 species clade in the order *Hyphomicrobiales* (syn. *Rhizobiales*) (25 of which encode SbmA/BacA) (Fig. 4). Interestingly, the SbmA/BacA proteins of the order *Enterobacterales* form a monophyletic group in the SbmA/BacA and BclA protein phylogeny (Fig. S3). On the other hand, the minimal monophyletic clade encompassing all *Hyphomicrobiales* SbmA/BacA proteins also includes the *Enterobacterales* SbmA/BacA proteins (Fig. S3). These results suggest that SbmA/BacA proteins of the order *Enterobacterales* may have been acquired through horizontal transfer from the order *Hyphomicrobiales*. The remaining 22 SbmA/BacA proteins not found within those two clades are distributed across the phylum *Pseudomonadota* with no other major clustering observed. Overall, these results suggest that although SbmA/BacA proteins are widespread amongst subclades of the orders *Enterobacterales* (class *Gammaproteobacteria*) and *Hyphomicrobiales* (class *Alphaproteobacteria*), the taxonomic distribution of this protein family is otherwise limited.

BclA proteins show a broader taxonomic distribution than the SbmA/BacA proteins, although their distribution remains restricted to only a few phyla (Fig. 4). Like SbmA/BacA, BclA is common in a subclade of the order *Hyphomicrobiales*, in which 19 of 21 species encoded BclA. Most of the other *Alphaproteobacteria* species encoding BclA belong to the order *Rhodospirillales*, in which 9 of the 30 species encode BclA. Within the *Gammaproteobacteria*, the taxon most enriched for BclA proteins is the order *Pasteurellales*, in which 10 of the 16 species encode BclA. BclA is also abundant in the class *Betaproteobacteria*, unlike SbmA/BacA, and is particularly enriched in the orders *Burkholderiales* (32/60 species) and *Neisseriales* (10/17 species) compared to the orders *Nitrosomonadales* and *Rhodocyclales* (4/27 species across both orders). In contrast to SbmA/BacA, which appears to be encoded only by species of the phylum *Pseudomonadota*, there are three main clades of organisms predicted to encode BclA outside of the phylum *Pseudomonadota* (Fig. 4). The largest of these are the phylum *Cyanobacteriota* (syn. *Cyanobacteria*), in which BclA is broadly distributed and found in 21 of the 31 species (68%). The other two main groups of organisms encoding BclA are a subclade of eight species (seven of which encode BclA) of the order *Mycobacteriales* [phylum *Actinomycetota* (syn. *Actinomycetes*)] and the class *Negativicutes* [phylum *Bacillota* (syn. *Firmicutes*)], in which seven of the ten species encode BclA homologues (Fig. 4).

## Discussion

We identified 71 SbmA/BacA and 177 BclA homologues from a search of the proteomes of 1,255 bacterial species. In total, 208 of the 1,255 species (16.6%) encoded at least one copy of SbmA/BacA and/or BclA, with only 6 of the 208 species (2.9%) encoding both SbmA/BacA and BclA. The observation that SbmA/BacA and BclA proteins were generally not encoded in the same proteome agrees with these protein families having similar biological roles. We also observed that the so-called ‘*Mycobacterium* BacA’ proteins clustered with the BclA proteins in both the SSN and the protein phylogeny, leading us to conclude that the ‘*Mycobacterium* BacA’ proteins are not distinct from BclA; we therefore reclassified the ‘*Mycobacterium* BacA’ proteins as BclA for downstream analyses.

### Evolution of the SbmA/BacA and BclA protein families

One of the objectives motivating this work was to examine the evolutionary histories of the SbmA/BacA and BclA protein families and, in particular, to gain insight into whether the SbmA/BacA and BclA protein families evolved independently or whether they share common ancestry (e.g. that SbmA/BacA evolved from BclA or vice versa). Our results suggest that the SbmA/BacA protein family likely originated in the order *Hyphomicrobiales* (syn. *Rhizobiales*) prior to spreading to other *Pseudomonadota* (syn. *Proteobacteria*) species via horizontal gene transfer. In addition, the taxonomic distribution of SbmA/BacA and BclA proteins within the order *Hyphomicrobiales* is suggestive of SbmA/BacA having evolved from an ancestral BclA protein. Excluding the deep-branching lineages, the order *Hyphomicrobiales* can be subdivided into two sister clades; SbmA/BacA is widely distributed in one of these clades, while BclA is widely distributed in the other, suggesting that the SbmA/BacA and BclA proteins of the order *Hyphomicrobiales* evolved from a common ancestral protein present in the ancestor of these clades. On the other hand, the *Hyphomicrobiales* SbmA/BacA and BclA proteins are polyphyletic in the protein phylogeny (Fig. 1), which could instead suggest that the SbmA/BacA and BclA proteins of the order *Hyphomicrobiales* were independently acquired. Nevertheless, we favour the more parsimonious explanation that SbmA/BacA evolved from an ancestral BclA protein within the order *Hyphomicrobiales*.

In total, 28 of the BclA proteins were encoded by 21 cyanobacteria. These 28 proteins formed a distinct cluster in the SSN together with 15 non-cyanobacterial BclA proteins, raising the possibility that these proteins evolved independently from the rest of the BclA proteins. While we cannot rule out this possibility, we consider the evidence to be insufficient to reach this conclusion at this time.

### The SbmA/BacA and BclA protein families are associated with eukaryotic host interaction

A second objective of this work was to determine how broadly SbmA/BacA and BclA proteins are distributed across the domain *Bacteria*. We note that our protein classification methodology relied on reference databases, and we are therefore limited to discussing the distribution of SbmA/BacA and BclA proteins in taxa that are represented in these databases, which may be biased towards over-representation of eukaryote-associated bacteria. Despite this limitation, the distribution of SbmA/BacA and BclA proteins amongst the known bacterial genomes we analysed revealed interesting trends. Contrary to our initial expectations, we found that both protein families display limited taxonomic distribution. SbmA/BacA homologues were identified only in the phylum *Pseudomonadota*, with 89% of the identified BacA proteins being encoded by species of the classes *Alphaproteobacteria* and *Gammaproteobacteria*. A majority of the identified BclA proteins were also found in species of the phylum *Pseudomonadota* with a bias towards the *Betaproteobacteria*; however, BclA proteins were also common in the phylum *Cyanobacteriota* (syn. *Cyanobacteria*), the class *Negativicutes* [phylum *Bacillota* (syn. *Firmicutes*)] and the order *Mycobacteriales* [phylum *Actinomycetota* (syn. *Actinomycetes*)]. Interestingly, many of the clades enriched for species encoding SbmA/BacA or BclA homologues also include many species known to interact with eukaryotic hosts in mutualistic or pathogenic interactions.

In total, 45 of the 55 species (82%) of the alphaproteobacterial order *Hyphomicrobiales* encode SbmA/BacA and/or BclA; this increases to 45 of 50 species (90%) when excluding the deep-branching *Hyphomicrobiales* lineages. This order accounts for 79% of the alphaproteobacterial species encoding SbmA/BacA and/or BclA homologues. Many members of the order *Hyphomicrobiales* are notable for their ability to interact with eukaryotic hosts. All alpha-rhizobia (legume mutualists) belong to the order *Hyphomicrobiales*, which also encompasses several plant and mammalian pathogens like *Agrobacterium* and *Brucella*, respectively [58]. Similarly, 75% of the gammaproteobacterial SbmA/BacA and BclA proteins are encoded by species in the orders *Enterobacterales* and *Pasteurellales*, in which 34 of 47 (72%; increasing to 81% when excluding a monophyletic group of five obligate endosymbionts) and 10 of 16 (62.5%) species encode SbmA/BacA or BclA, respectively. The order *Enterobacterales* is well-known for including many plant (e.g. *Dickeya* and *Pantoea*) and animal/human (e.g. *Klebsiella* and *Yersinia*) pathogens [59]. Likewise, the order *Pasteurellales* encompasses several animal/human pathogens (e.g. *Haemophilus* and *Pasteurella*) [60]. In the class *Betaproteobacteria*, BclA and SbmA/BacA were significantly more common in the orders *Burkholderiales* and *Neisseriales* compared to the orders *Nitrosomonadales* and *Rhodocyclales*. The order *Burkholderiales* encompasses all known beta-rhizobia (legume mutualists) as well as insect gut symbionts (e.g. *Caballeronia*) and plant (e.g. *Ralstonia*) and animal/human (e.g. *Burkholderia*) pathogens [61,62]. The order *Neisseriales* encompasses many mammalian commensals but also some human pathogens (e.g. *Neisseria*) [63].

The phylum *Cyanobacteria* is the largest clade of organisms encoding BclA proteins outside of the phylum *Pseudomonadota*. To our knowledge, cyanobacteria are not pathogenic. However, many can form beneficial associations with diverse hosts, such as the nitrogen-fixing symbiosis between *Nostoc* and plants [64], the mutualistic relationship with fungi (forming lichens) and with sponges [65]. The order *Mycobacteriales* includes important human and plant pathogens (e.g. *Mycobacterium* and *Rhodococcoides*) [66] and opportunistic pathogens (e.g. *Mycolicibacterium*) [67]. The class *Negativicutes* is poorly studied despite its peculiar nature, as these *Bacillota* possess an outer membrane and lipopolysaccharide [68]. Nevertheless, this class is a common component of eukaryotic microbiomes and can cause human disease, including meningitis [69].

The observation that most taxonomic clades enriched for species encoding SbmA/BacA or BclA also contain many mutualistic and/or pathogenic organisms may suggest that eukaryotic host interaction is a driver of SbmA/BacA and BclA maintenance in these lineages. However, the data also suggest that these protein families may predate these species interactions. Assuming that SbmA/BacA originated in the common ancestor of the SbmA/BacA-containing subclade of the order *Hyphomicrobiales*, the SbmA/BacA protein family potentially evolved in this lineage over 500 million years ago [70], which predates the evolution of legumes that are estimated to have evolved around 60 million years ago [71]. Thus, SbmA/BacA could not have evolved in this lineage as a response to legume symbiosis. Rather, we hypothesize that SbmA/BacA originally evolved to fulfil another role (such as nutrient transport [17] or protection against membrane-damaging AMPs produced by microbial competitors [72]) and was subsequently co-opted to support legume symbiosis in rhizobia. Likewise, we hypothesize that BclA already existed in the *Bradyrhizobium* lineage prior to the evolution of legume symbiosis and that this protein was independently co-opted for legume symbiosis in these organisms, mimicking the convergent evolution of NCR peptides in the inverted repeat lacking clade and Dalbergioid legume families [26]. On the other hand, the absence of SbmA/BacA and BclA proteins in most bacterial lineages may reflect that these proteins also sensitize bacteria to AMPs with intracellular targets, resulting in a fitness disadvantage in inter- and intraspecific competition. For example, phazolicin is a narrow-spectrum AMP that is produced by some rhizobial strains and that can kill other rhizobia after being imported by BacA and YejABEF transporters [18]. Likewise, microcins J25 and B17 are narrow-spectrum AMPs produced by *E. coli* that depend on SbmA/BacA to reach their intracellular targets in target organisms [73]. Considering this, we hypothesize that SbmA/BacA and BclA have been selected for in bacteria where resistance to membrane-targeting AMPs is more important than resistance to AMPs with intracellular targets, such as during host interaction, where these proteins may have been repeatedly co-opted to help bacteria survive exposure to host-encoded AMPs. The absence of these proteins would be favoured when bacteria predominantly encounter AMPs with intracellular targets, which may be the case for non-host-associated microbes primarily encountering AMPs produced by other microbes.

### Transport of AMPs is a general property of SbmA/BacA and BclA proteins

The abilities of several newly identified BclA proteins to complement the phenotypes of an *S. meliloti* ∆*bacA* mutant were tested to validate that our bioinformatic pipeline correctly identified functionally similar proteins. *S. meliloti bacA* null mutants display increased Gm resistance compared to the wild type [74]. Eight of the nine synthesized genes at least partially complemented the Gm resistance phenotype of an *S. meliloti* ∆*bacA* mutant, suggesting these eight proteins were expressed and at least partially functional in *S. meliloti*. Interestingly, even the gene encoding an ExsE protein partially complemented the Gm resistance phenotype, indicating that Gm transport is not specific to SbmA/BacA and BclA proteins but is a general property of these and related protein families. Gm sensitivity assays are commonly used to characterize the function of rhizobial *bacA* proteins and rhizobial *bacA* mutant alleles generated through site-directed mutagenesis [20]. Although these assays are useful to identify null phenotypes, our results show that they do not probe a function unique to SbmA/BacA or BclA proteins and thus have limited value as a proxy to peptide transport or host interaction assays.

In addition to showing increased resistance to Gm, *S. meliloti* ∆*bacA* mutants show increased sensitivity to NCR peptides [14,20]. As the antimicrobial activity of NCR peptides is a result of their interaction with the cell envelope, it is thought that SbmA/BacA and BclA proteins provide resistance to NCR peptides by moving the peptides into the cell and thus away from the cell envelope [14,32]. SbmA/BacA and BclA proteins have also been shown to transport other AMPs, including mammalian peptides such as Bac7 [7,8, 13, 18]. Only the proteins annotated as BclA were capable of effectively complementing the sensitivity of *S. meliloti* ∆*bacA* and *S. meliloti* ∆*bacA* Ω*yejA* mutants to the NCR peptide NCR247. Of the six newly identified BclA proteins that were tested, four repeatedly demonstrated good levels of complementation; these proteins were from *P. naphthalenivorans* (class *Betaproteobacteria*), *S. dextrinosolvens* (class *Gammaproteobacteria*), *S. elongatus* (phylum *Cyanobacteriota*) and *C. aponinum* (phylum *Cyanobacteriota*). The other two, from *M. anaerophila* (class *Negativicutes*) and *B. psittacipulmonis* (class *Betaproteobacteria*), showed weak and variable or little to no complementation, respectively. However, there are thousands of distinct NCR peptides encoded across the legume family [29], and thus the inability of any given protein to transport NCR247 does not necessarily mean that it is unable to transport other NCR peptides or mammalian AMPs. Indeed, *S. meliloti yejA* mutants show increased sensitivity to the peptide NCR280 but not NCR247 [23]. Regardless, these results support that the ability to transport AMPs, including NCR peptides, is a general property of bacterial SmbA/BacA and BclA proteins.

## Conclusions

In summary, our analyses suggest that SbmA/BacA likely evolved from an ancestral BclA protein early in the evolution of the bacterial order *Hyphomicrobiales* (syn. *Rhizobiales*). Overall, we identified 208 bacterial species encoding SbmA/BacA or BclA. These species were not equally distributed across the domain *Bacteria*; instead, SbmA/BacA proteins were found only in the phylum *Pseudomonadota*, while BclA proteins were primarily found within a subset of families across four phyla. Our analyses suggest that the SbmA/BacA and BclA protein families have been repeatedly co-opted to facilitate both mutualistic and pathogenic associations with eukaryotic hosts by allowing bacteria to cope with host-encoded AMPs. We further suggest that the distribution of SbmA/BacA and BclA is determined by the fitness trade-off associated with their presence. Specifically, we predict that genes encoding SbmA/BacA or BclA will only be maintained in bacteria for which resistance to membrane-targeting AMPs is more important than resistance to AMPs with intracellular targets.

## Supplementary material

10.1099/mgen.0.001380 Supplementary Material 1.

10.1099/mgen.0.001380 Supplementary Data Sheet 1.

10.1099/mgen.0.001380 Supplementary Data Sheet 2.

## Abbreviations

AMPs: antimicrobial peptides
BIC: Bayesian information criterion
EFI-EST: enzyme function initiative’s enzyme similarity tool
Gm: gentamicin
HMMs: hidden Markov models
LB: lysogeny broth
NCBI: National Center for Biotechnology Information
NCR: nodule-specific cysteine-rich
SH-aLRT: Shimodaira–Hasegawa-like approximate likelihood ratio test
SSN: sequence similarity network
YEB: yeast extract beef
