# Supplementary material for: Taxonomic distribution of SbmA/BacA and BacA-like antimicrobial peptide transporters suggests independent recruitment and convergent evolution in host–microbe interactions
Source: Microb Genom. 2025 Apr 16;11(4):001380. doi: 10.1099/mgen.0.001380 (PMC12003926; doi:10.1099/mgen.0.001380)
Supplement: Supplementary Material 1. [file mgen-11-01380-s001.pdf]

Supplementary Material for
Taxonomic distribution of SbmA/BacA and BacA-like antimicrobial peptide transporters suggests independent recruitment and convergent evolution in host-microbe interactions

Nicholas T. Smith+, Amira Boukherissa+, Kiera Antaya, Graeme W. Howe, Peter Mergaert, Ricardo C Rodríquez de la Vega, Jacqui A. Shykoff, Benoît Alunni*, George C. diCenzo*
+ N.T.S. and A.B. contributed equally to this work.

Benoît Alunni
Email: benoit.alunni@inrae.fr

George C. diCenzo
Email: george.dicenzo@queensu.ca

This PDF file includes:

Tables S1 to S2 (pages 2-4)
Figures S1 to S4 (pages 5-8)
Legends for Datasets S1 to S2 (page 9)

Other supplementary materials for this manuscript include the following:

Datasets S1 to S2

Table S1. Shoot dry weights of legumes inoculated with a *Sinorhizobium meliloti* Δ*bacA* mutant and *S. meliloti* Δ*bacA* mutants expressing various *sbmA*-like proteins *in trans*.

***S. meliloti* genotype ***	**Mean shoot dry weight ± standard deviation (mg/plant)**
***Medicago sativa* (alfalfa)**	
Wildtype Rm1021	66.7 ± 7.1
Δ*bacA* empty vector control	7.6 ± 1.1
Sinorhizobium meliloti 1021 *bacA*	68.6 ± 4.9
Polymorphum gilvum bacA	8.8 ± 1.7
Bradyrhizobium sp. ORS285 *bclA*	7.1 ± 0.6
Succinivibrio dextrinosolvens bclA	7.7 ± 1.1
Basilea psittacipulmonis bclA	10.7 ± 1.2
Polaromonas naphthalenivorans bclA	8.8 ± 0.8
Synechococcus elongatus bclA	6.1 ± 0.9
Methylomusa anaerophila bclA	8 ± 1.4
Cyanobacterium aponinum bclA	7.1 ± 0.2
Eikenella exigua bclA-like	6.7 ± 1.3
Phyllobacterium zundukense exsE	7.7 ± 1.2
Uninoculated control	6.5 ± 1.5
***Melilotus officinalis* (yellow-blossom sweet clover)**	
Wild type Rm1021	72.2 ± 4.5
Δ*bacA* empty vector control	6.8 ± 1.3
Sinorhizobium meliloti 1021 *bacA*	35.6 ± 8.7
Polymorphum gilvum bacA	5.0 ± 0.6
Bradyrhizobium sp. ORS285 *bclA*	3.1 ± 0.7
Succinivibrio dextrinosolvens bclA	6.3 ± 1.3
Basilea psittacipulmonis bclA	5.2 ± 0.2
Polaromonas naphthalenivorans bclA	3.5 ± 1.9
Synechococcus elongatus bclA	6.8 ± 1.9
Methylomusa anaerophila bclA	6.9 ± 0.6
Cyanobacterium aponinum bclA	4.6 ± 0.2
Eikenella exigua bclA-like	7.2 ± 0.4
Phyllobacterium zundukense exsE	3.6 ± 0.2
Uninoculated	6 ± 1.1

* Strains included the wildtype *S. meliloti* strain Rm1021, a Rm1021 Δ*bacA* derivative, and Δ*bacA* derivatives expressing the genes from the indicated organisms *in trans*.

Table S2. Bacterial strains and plasmids

Strain/Plasmid	Characteristics	Reference
Sinorhizobium meliloti		
Rm1021	Wild type SU47 *str-21*; Sm^{R}	Meade *et al.* (1982)
SmGF1	Rm1021 *bacA*654::Spc (Δ*bacA* null); Sm^{R} Sp^{R}	Ferguson *et al.* (2002)
Rm1021Δ*bacA*Ω*yejA*	Rm1021 Δ*bacA* Ω*yejA*; Sm^{R} Sp^{R} Km^{R}	Travin DY *et al.* (2023)
SmGQ0019	SmGF1 (pGQ0037); Sm^{R} Sp^{R} Tc^{R}	This study
SmGQ0020	SmGF1 (pGQ0038); Sm^{R} Sp^{R} Tc^{R}	This study
SmGQ0021	SmGF1 (pGQ0039); Sm^{R} Sp^{R} Tc^{R}	This study
SmGQ0022	SmGF1 (pGQ0040); Sm^{R} Sp^{R} Tc^{R}	This study
SmGQ0023	SmGF1 (pGQ0041); Sm^{R} Sp^{R} Tc^{R}	This study
SmGQ0024	SmGF1 (pGQ0042); Sm^{R} Sp^{R} Tc^{R}	This study
SmGQ0025	SmGF1 (pGQ0043); Sm^{R} Sp^{R} Tc^{R}	This study
SmGQ0026	SmGF1 (pGQ0044); Sm^{R} Sp^{R} Tc^{R}	This study
SmGQ0027	SmGF1 (pGQ0045); Sm^{R} Sp^{R} Tc^{R}	This study
SmGQ0028	SmGF1 (pGQ0046); Sm^{R} Sp^{R} Tc^{R}	This study
SmGQ0029	SmGF1 (pRF771-*bclA*-ORS285); Sm^{R} Sp^{R} Tc^{R}	This study
SmGQ0030	SmGF1 (pRF771); Sm^{R} Sp^{R} Tc^{R}	This study
SmGQ0031	SmGF1 (pRF771-*bacA*-Rm1021); Sm^{R} Sp^{R} Tc^{R}	This study
SmGQ0032	SmGF1 (pRF771-*bclA*-USDA110); Sm^{R} Sp^{R} Tc^{R}	This study
SmYjaGQ0019	Rm1021Δ*bacA*Ω*yejA* (pGQ0037); Sm^{R} Tc^{R} Sp^{R} Km^{R}	This study
SmYjaGQ0020	Rm1021Δ*bacA*Ω*yejA* (pGQ0038); Sm^{R} Tc^{R} Sp^{R} Km^{R}	This study
SmYjaGQ0021	Rm1021Δ*bacA*Ω*yejA* (pGQ0039); Sm^{R} Tc^{R} Sp^{R} Km^{R}	This study
SmYjaGQ0022	Rm1021Δ*bacA*Ω*yejA* (pGQ0040); Sm^{R} Tc^{R} Sp^{R} Km^{R}	This study
SmYjaGQ0023	Rm1021Δ*bacA*Ω*yejA* (pGQ0041); Sm^{R} Tc^{R} Sp^{R} Km^{R}	This study
SmYjaGQ0024	Rm1021Δ*bacA*Ω*yejA* (pGQ0042); Sm^{R} Tc^{R} Sp^{R} Km^{R}	This study
SmYjaGQ0025	Rm1021Δ*bacA*Ω*yejA* (pGQ0043); Sm^{R} Tc^{R} Sp^{R} Km^{R}	This study
SmYjaGQ0026	Rm1021Δ*bacA*Ω*yejA* (pGQ0044); Sm^{R} Tc^{R} Sp^{R} Km^{R}	This study
SmYjaGQ0027	Rm1021Δ*bacA*Ω*yejA* (pGQ0045); Sm^{R} Tc^{R} Sp^{R} Km^{R}	This study
SmYjaGQ0028	Rm1021Δ*bacA*Ω*yejA* (pGQ0046); Sm^{R} Tc^{R} Sp^{R} Km^{R}	This study
SmYjaGQ0029	Rm1021Δ*bacA*Ω*yejA* (pRF771-*bclA*-ORS285); Sm^{R} Tc^{R} Sp^{R} Km^{R}	This study
SmYjaGQ0030	Rm1021Δ*bacA*Ω*yejA* (pRF771); Sm^{R} Tc^{R} Sp^{R} Km^{R}	This study
SmYjaGQ0031	Rm1021Δ*bacA*Ω*yejA* (pRF771-*bacA*-Rm1021); Sm^{R} Tc^{R} Sp^{R} Km^{R}	This study
SmYjaGQ0032	Rm1021Δ*bacA*Ω*yejA* (pRF771-*bacA*-USDA110); Sm^{R} Tc^{R} Sp^{R} Km^{R}	This study
Escherichia coli		
MT616	MM294A *recA-56* (pRK600), mobilizer; Cm^{R}	Finan *et al.* (1986)
HB101	F–, *thi*-1, *hsdS*20 (r_B^-, m_B^-), *supE*44, *recA*13, *ara*-14, *leuB*6, *proA*2, *lacY*1, *galK*2, *rpsL*20, *xyl*-5, *mtl*-1; Sm^{R}	Boyer & Roulland-Dussoix (1969)
HB101.pRK600	HB101 (pRK600), mobilizer; Cm^{R}	Laboratory collection
**Plasmids**		
pRF771	pTE3 derivative with *trp* promoter; Tc^{R}	Wells and Long (2002)
pRF771-*bacA*-Rm1021	pRF771-*Sinorhizobium meliloti* Rm1021 *bacA*; Tc^{R}	Haag *et al.* (2011)
pRF771-*bclA*-ORS285	pRF771-*Bradyrhizobium* sp. ORS285 *bclA*; Tc^{R}	Guefrachi *et al.* (2015)
pRF771-*bclA*-USDA110	pRF771-*Bradyrhizobium diazoefficiens* USDA110 *bclA*, Tc^{R}	Barrière *et al.* (2017)
pGQ0037	pRF771-*Succinivibrio dextrinosolvens bclA*; Tc^{R}	This study
pGQ0038	pRF771-*Polymorphum gilvum bacA*; Tc^{R}	This study
pGQ0039	pRF771-*Basilea psittacipulmonis bclA*; Tc^{R}	This study
pGQ0041	pRF771-*Polaromonas naphthalenivorans bclA*; Tc^{R}	This study
pGQ0042	pRF771-*Phyllobacterium zundukense exsE*; Tc^{R}	This study
pGQ0043	pRF771-*Eikenella exigua bclA*-like gene; Tc^{R}	This study
pGQ0044	pRF771-*Synechococcus elongatus bclA*; Tc^{R}	This study
pGQ0045	pRF771-*Methylomusa anaerophila bclA*; Tc^{R}	This study
pGQ0046	pRF771-*Cyanobacterium aponinum bclA*; Tc^{R}	This study

Sm – streptomycin; Sp – spectinomycin; Km – kanamycin; Tc – tetracycline; Cm – chloramphenicol

Barrière, Q., Guefrachi, I., Gully, D., Lamouche, F., Pierre, O., Fardoux, J., Chaintreuil, C., Alunni, B., Timchenko, T., Giraud, E., and Mergaert, P. 2017. Integrated roles of BclA and DD-carboxypeptidase 1 in *Bradyrhizobium* differentiation within NCR-producing and NCR-lacking root nodules. Sci Rep **7**(1): 9063. doi:10.1038/s41598-017-08830-0.

Boyer, H.W., and Roulland-dussoix, D. 1969. A complementation analysis of the restriction and modification of DNA in *Escherichia coli*. J Mol Biol **41**(3): 459–472. doi:10.1016/0022-2836(69)90288-5.

Ferguson, G.P., Roop, R.M., and Walker, G.C. 2002. Deficiency of a *Sinorhizobium meliloti bacA* mutant in alfalfa symbiosis correlates with alteration of the cell envelope. J Bacteriol **184**(20): 5625–5632. doi:10.1128/JB.184.20.5625-5632.2002.

Finan, T.M., Kunkel, B., De Vos, G.F., and Signer, E.R. 1986. Second symbiotic megaplasmid in *Rhizobium meliloti* carrying exopolysaccharide and thiamine synthesis genes. J Bacteriol **167**(1): 66–72.

Guefrachi, I., Pierre, O., Timchenko, T., Alunni, B., Barrière, Q., Czernic, P., Villaécija-Aguilar, J.-A., Verly, C., Bourge, M., Fardoux, J., Mars, M., Kondorosi, E., Giraud, E., and Mergaert, P. 2015. *Bradyrhizobium* BclA is a peptide transporter required for bacterial differentiation in symbiosis with *Aeschynomene* legumes. Mol Plant Microbe Interact **28**(11): 1155–1166. doi:10.1094/MPMI-04-15-0094-R.

Haag, A.F., Baloban, M., Sani, M., Kerscher, B., Pierre, O., Farkas, A., Longhi, R., Boncompagni, E., Hérouart, D., Dall'Angelo, S., Kondorosi, E., Zanda, M., Mergaert, P., and Ferguson, G.P. 2011. Protection of *Sinorhizobium against* host cysteine-rich antimicrobial peptides is critical for symbiosis. PLOS Biol **9**(10): e1001169. doi:10.1371/journal.pbio.1001169.

Meade, H.M., Long, S.R., Ruvkun, G.B., Brown, S.E., and Ausubel, F.M. 1982. Physical and genetic characterization of symbiotic and auxotrophic mutants of *Rhizobium meliloti* induced by transposon Tn*5* mutagenesis. J Bacteriol **149**(1): 114–122.

Travin, D.Y., Jouan, R., Vigouroux, A., Inaba-Inoue, S., Lachat, J., Haq, F., Timchenko, T., Sutormin, D., Dubiley, S., Beis, K., Moréra, S., Severinov, K., and Mergaert, P. 2023. Dual-uptake mode of the antibiotic phazolicin prevents resistance acquisition by Gram-negative bacteria. mBio **14**(2): e0021723. doi:10.1128/mbio.00217-23.

Wells, D.H., and Long, S.R. 2002. The *Sinorhizobium meliloti* stringent response affects multiple aspects of symbiosis. Mol Microbiol **43**(5): 1115–1127. doi:10.1046/j.1365-2958.2002.02826.x.

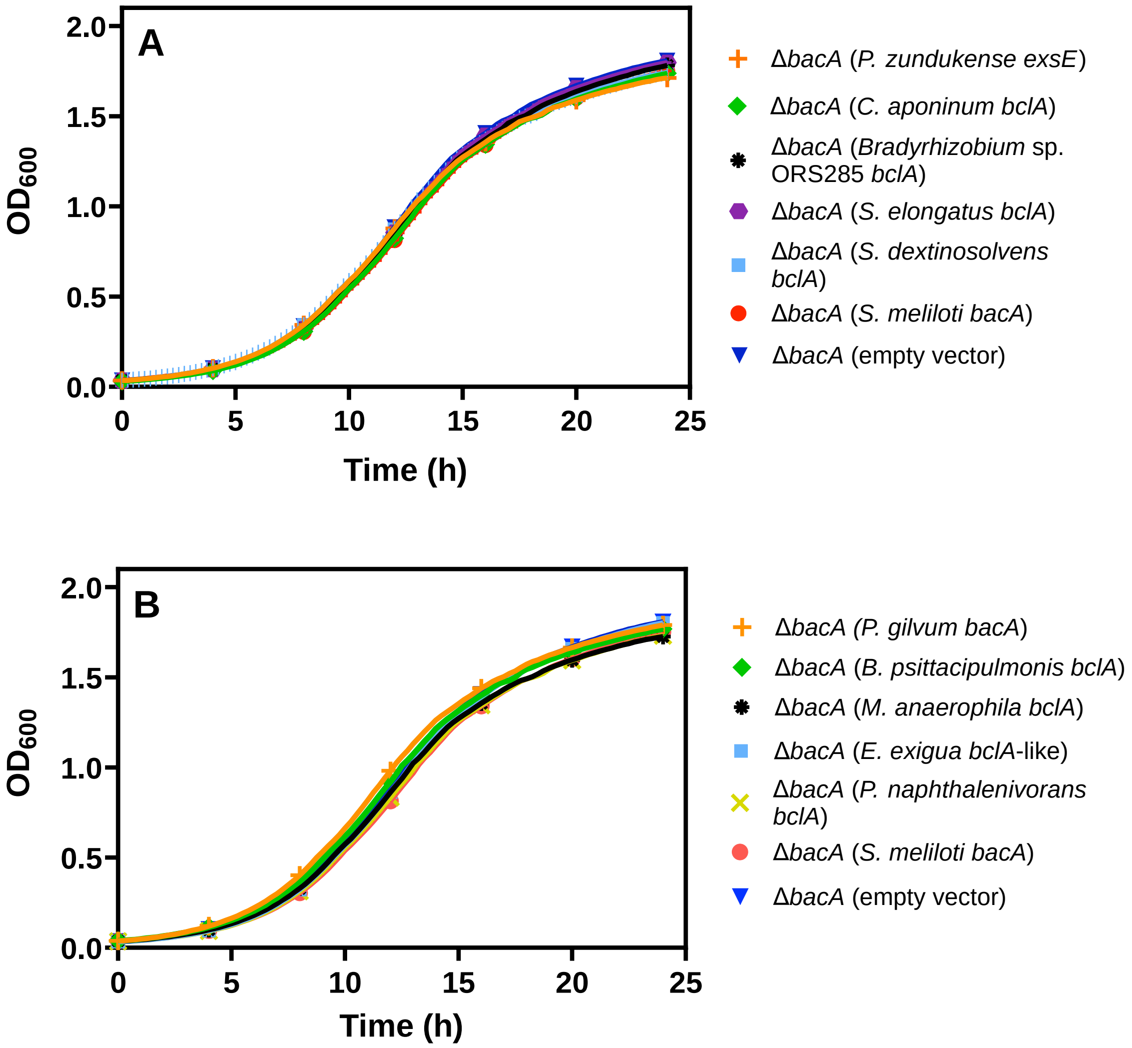

Figure S1. Growth of *Sinorhizobium meliloti* strains in LBmc. The growth of various *S. meliloti* strains, as measured by OD600, in LBmc is shown over a 24-hour period. Each point represents the mean of triplicate wells, with error bars depicting standard deviation. The Δ*bacA* strain represents the *S. meliloti* Δ*bacA* mutant carrying an empty vector, while all other strains are named according to the species of origin of the gene expressed *in trans* in the *S. meliloti* Δ*bacA* background. The experiment was replicated three independent times, and data from a representative experiment is shown.

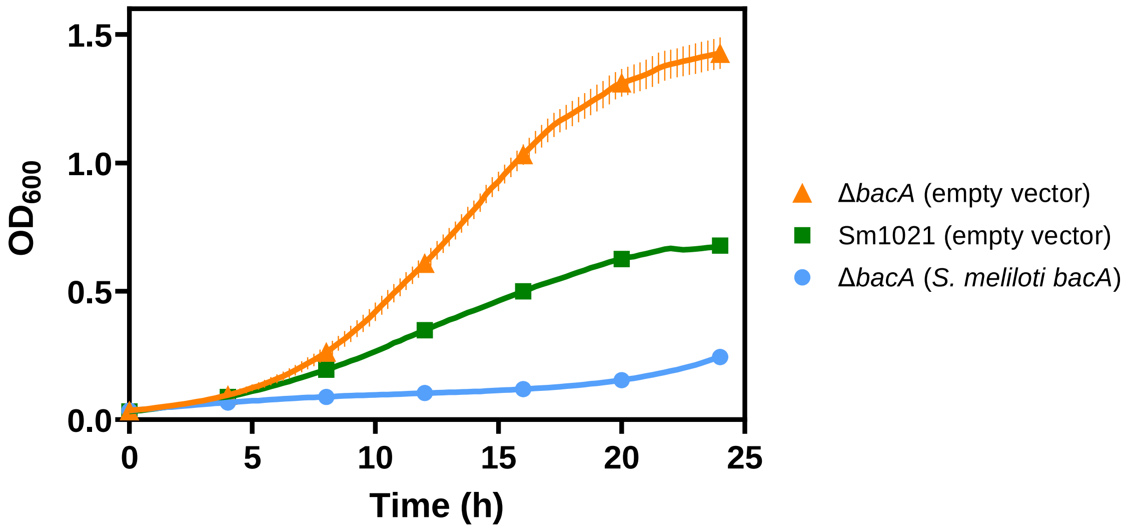

Figure S2. Effect of expressing *bacA in trans* on the gentamicin sensitivity of *Sinorhizobium meliloti*. The growth of three *S. meliloti* strains, as measured by OD600, in the presence of 20 µg/mL of gentamicin is shown over a 24-hour period. Growth profiles are shown for wildtype *meliloti* Rm1021 harbouring an empty expression vector (blue), and a *S. meliloti* Δ*bacA* mutant expressing (blue) or not (orange) the *S. meliloti bacA* gene *in trans*. Each point represents the mean of triplicate wells, with error bars depicting standard deviation. The experiment was replicated three independent times, and data from a representative experiment is shown.

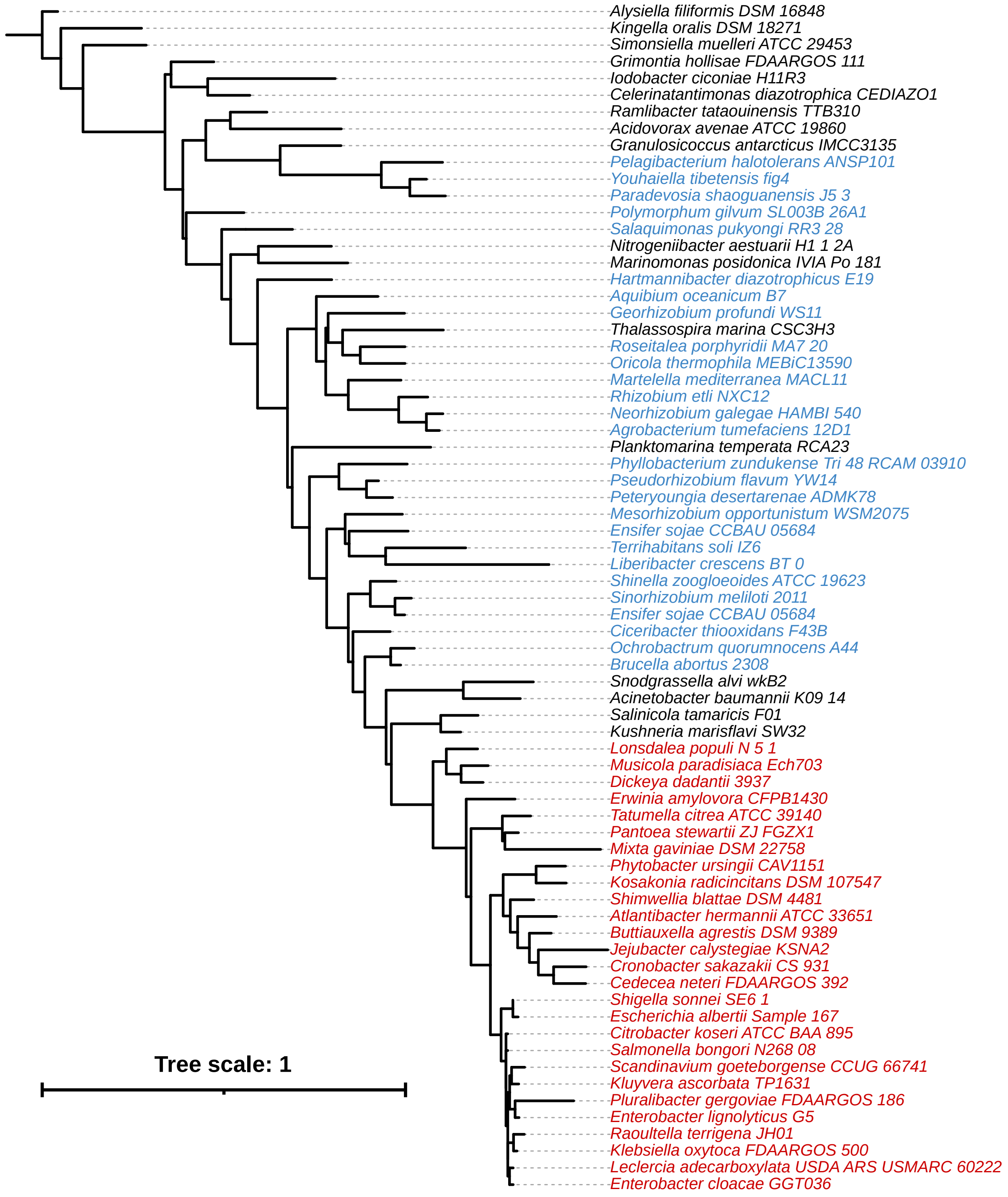

Figure S3. Maximum-likelihood phylogeny of SbmA/BacA proteins. A subtree of the maximum likelihood phylogeny of SbmA/BacA-like proteins of Figure 1 is shown. This subtree is limited to the 71 proteins classified as BacA. Protein encoded by species of the order *Enterobacterales* are shown in red, while proteins of the order *Hyphomicrobiales* are shown in blue. The scale bar represents the average number of amino acid substitutions per site. An interactive version of this phylogeny, with node support values, is provided through iTol (https://itol.embl.de/shared/1IAjjFrHYGLI9).

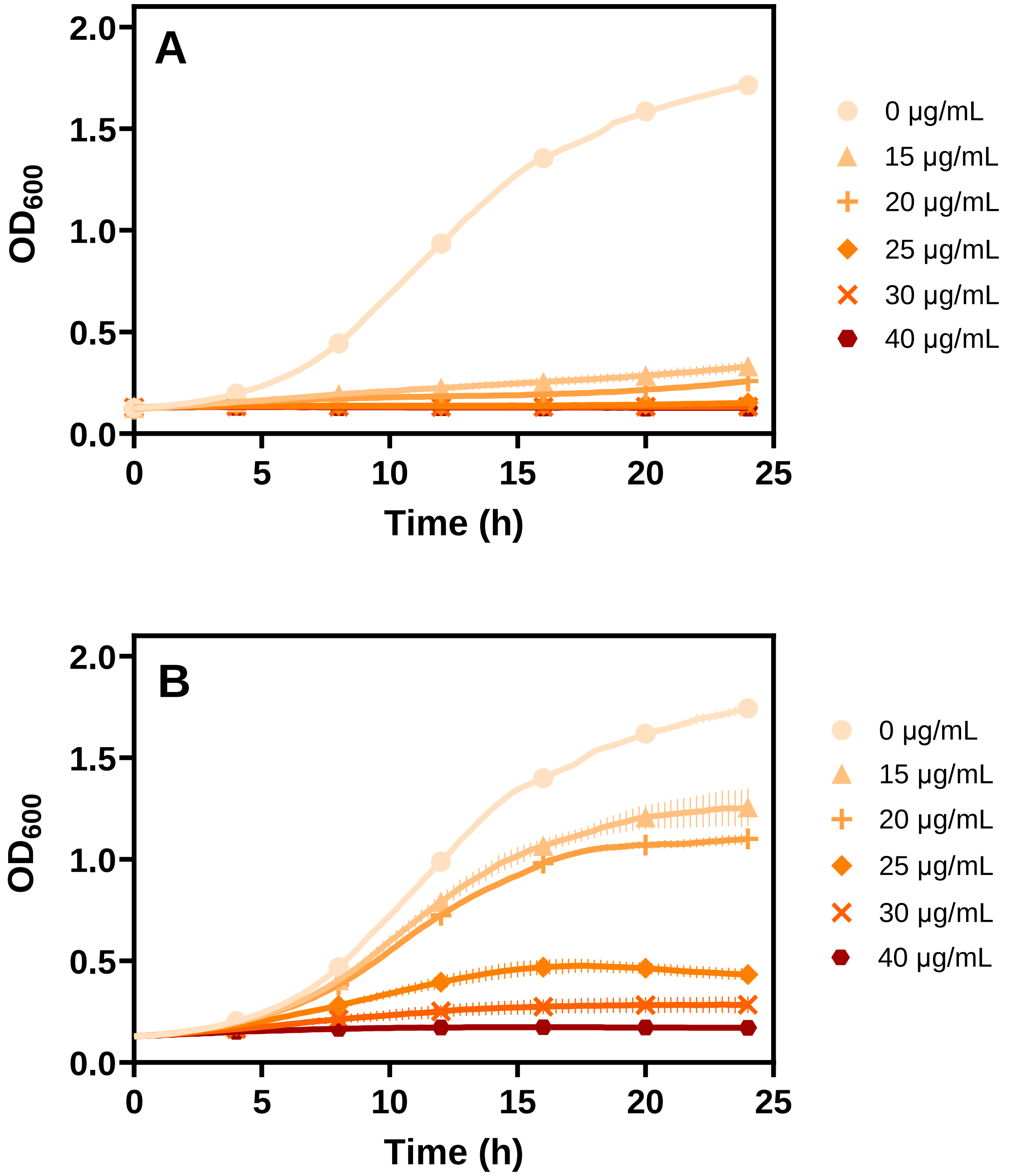

Figure S4. Effect of gentamicin on the growth of *Sinorhizobium meliloti*. The growth of *S. meliloti* in LBmc containing various concentrations of gentamicin, as indicated in the legend. Growth profiles are shown for (**A**) *S. meliloti* Δ*bacA* carrying an empty expression vector, and (**B**) *S. meliloti* Δ*bacA* expressing the *S. meliloti bacA* gene *in trans*. Each point represents the mean of triplicate wells, with error bars depicting standard deviation. The experiment was replicated three independent times, and data from a representative experiment is shown.

DATASET LEGENDS

Dataset S1. Metadata for the 1,255 bacterial genomes used in this study, including ftp links to download the genomes from the National Center for Biotechnology Information (NCBI) database. **(tab-delimited)**

Dataset S2. The sequences, locus tag, organism or origin, HMM classification, SSN cluster, and final classification for the 366 proteins included in the phylogeny of Figure 1. The first column indicates the nine genes that were codon optimized for *Sinorhizobium meliloti* and synthesized. **(tab-delimited)**
